# A Mode Matched Triaxial Vibratory Wheel Gyroscope with Fully Decoupled Structure

**DOI:** 10.3390/s151128979

**Published:** 2015-11-17

**Authors:** Dunzhu Xia, Lun Kong, Haiyu Gao

**Affiliations:** School of Instrument Science and Engineering, Key Laboratory of Micro Inertial Instrument and Advanced Navigation Technology of the Ministry of Education, Southeast University, Nanjing 210096, China; E-Mails: konglun_2015@163.com (L.K.); 101010203@seu.edu.cn (H.G.)

**Keywords:** triaxial gyroscope, gyroscope structure, artificial fish swarm algorithm, mode matching

## Abstract

To avoid the oscillation of four unequal masses seen in previous triaxial linear gyroscopes, a modified silicon triaxial gyroscope with a rotary wheel is presented in this paper. To maintain a large sensitivity and suppress the coupling of different modes, this novel gyroscope structure is designed be perfectly symmetrical with a relatively large size of about 9.8 mm × 9.8 mm. It is available for differentially detecting three-axis angular rates simultaneously. To overcome the coupling between drive and sense modes, numerous necessary frames, beams, and anchors are delicately figured out and properly arranged. Besides, some frequency tuning and feedback mechanisms are addressed in the case of post processing after fabrication. To facilitate mode matched function, a new artificial fish swarm algorithm (AFSA) performed faster than particle swarm optimization (PSO) with a frequency split of 108 Hz. Then, by entrusting the post adjustment of the springs dimensions to the finite element method (FEM) software ANSYS, the final frequency splits can be below 3 Hz. The simulation results demonstrate that the modal frequencies in drive and different sense modes are respectively 8001.1, 8002.6, 8002.8 and 8003.3 Hz. Subsequently, different axis cross coupling effects and scale factors are also analyzed. The simulation results effectively validate the feasibility of the design and relevant theoretical calculation.

## 1. Introduction

With the development of MEMS technology, many categories of silicon micro-gyroscopes have been developed as key inertial sensors to detect angular rates. Benefitting from the advantages of light weight, compact size, low power, low cost and the potential for batch production, they have been widely used in industrial and consumer electronics applications [1,2,3]. Mostly, the researchers have focused on the improvement of single axis gyroscope performance, especially in the so called *z*-axis gyroscope. Considering that a monolithic triaxial gyroscope design is more competitive, despite its complexity, it is becoming a future development trend to easily form inertial measurement units [4].

To sense different axis rotation, we need drive in one direction and detect the response in another orthogonal direction according to the Coriolis effect. As we know, for a single axis gyroscope, only the in-plane drive and sense method is enough to sense *z*-axis rotation. However, to make a triaxial functional gyroscope, a lateral-axis gyroscope has been proposed and realized. To realize a lateral single axis gyroscope, an *x*-axis gyroscope with vertical drive and in-plane sensing was first proposed in 2005 [5]. Similarly, in 2010, a lateral-axis silicon micromachined tuning fork gyroscope was successfully designed [6]. The proposed gyroscope has lateral drive and torsional *z*-sensing with a decoupling function. Afterwards, the same group went on to propose a novel lateral-axis wheel gyroscope in 2011 [7]. The above developed gyroscope for only lateral-axis angular rate detection has driven the triaxial gyroscope revolution. Most importantly, the design is fully compatible with the fabrication process of *z*-axis gyroscopes.

In the commercial and academic fields, triaxial gyroscopes are always realized by two approaches. One is to assemble three orthogonal single-axis gyroscopes together [8]. Following this idea, the reliability assessment on a triaxial gyroscope under various shock loading conditions has been presented accordingly [9]. In this case, the gyroscopes used are realized by triple *z*-axis ones which are assembled together in a reciprocally orthogonal disposition. A monolithic triaxial silicon gyroscope with double *x*/*y*-axes and single *z*-axis configuration was investigated in 2013 [10]. Due to the limited assembly precision without a common mass center, a triaxial cross error among triaxial will be induced to cause further navigation errors in the IMU. On the contrary, if the alignment error can be effectively avoided or greatly decreased by a monolithic strategy, another approach is then proposed to achieve a similar precision though it has more challenging structure design requirements. It is very inspiring that some leading companies such as STMicroelectronics have made a commercial monolithic triaxial gyroscope with a single mass scheme that actually fully utilized a triple single-axis gyroscope combination [11]. From the measurement results, though the cross-axis errors are not greatly suppressed, it has led to successful applications in consumer electronics. Different from linear vibration gyroscopes, a new monolithic wheel type triaxial gyroscope is successfully fabricated and tested [12]. Both the *x-* and *y-* axis angular rates can be detected synchronously via out-of-plane plates, whereas the *z*-axis measurement is performed by in-plane comb fingers. Further, another delicate design of monolithic triaxial gyroscope is proposed [13]. By an ingenious spring design, the inner masses’ vibration is first linearly excited and transferred to a rotary motion with out-plane motion of special four proof masses, thus three axis detection can be achieved simultaneously.

Cross coupling between drive and sense modes in a single axis gyroscope is key issue. To improve the cross coupling effect in triaxial designs, a decoupled structure with drive to sense decoupling folded springs is designed and verified [14]. Since then, numerous decoupling structures were widely adopted by researchers. In particular, a single axis gyroscope with fully symmetrical decoupled style is proposed to realize the bidirectional decoupling between drive and sense modes [15]. Moreover, a silicon MEMS quadruple mass gyroscope with a perfectly symmetric decoupling structure is proposed, resulting in a lot of advantages such as matched modes, high quality factor and good temperature characterization [16]. Inspired by the above designs, all these merits are utilized here to propose a fully symmetric decoupling triaxial gyroscope structure. In the specific design details, since mode matching is essential to further improve the sensitivity of a triaxial gyroscope, therefore, a fully decoupled triaxial linear vibratory gyroscope with matched modes is proposed [17].

Using the ANSYS software, we found that the four masses have their own independent movements and this causes lots of interference modes. Especially, the position error between the main U shaped springs and big frames will easily cause undesired rotation modes. Though we can implement an ideal design in simulation, the inevitable fabrication errors always cause undesired results, such as the unequal vibration frequency and amplitude in drive modes. In consideration of imperfect problems, we have to merge these four proof masses and make them move synchronously and identically. For simplicity, a preferred embodiment of a triaxial vibratory wheel gyroscope is presented here. At the same time, the decoupling mechanism is still preserved. In fact, the transplantation from linear geometry to rotational geometry needs more complex and precision calculation. In the Lagrangian equations the general coordinates should be changed from displacement to angle, and all the terms should be changed from forces to torques. The different frames placement and springs design still needs more carefully optimization and calculations under a work load.

For a triple single-axis scheme, the vacuum encapsulation process will be performed three times in such a packaged triaxial gyroscope, accordingly the expense will be triple. The assembly process of three *z*-axis gyroscopes on a PCB is also three times as complex. Moreover, its performance as an IMU sensor is restricted by the inevitable alignment errors during the assembly. Besides, the circuits in drive mode will be three times as complex. Compared to triple orthogonal single-axis gyroscopes which are combined together, the proposed monolithic triaxial gyroscope will be fabricated at a considerably lower cost and in a small volume. As known to all, the trend of gyroscope development has been toward high integration density, which has been witnessed by many leading MEMS gyroscope manufacturers [1,4,7].

## 2. Wheel Type Triaxial Gyroscope

### 2.1. Fully Decoupled Structure

**Figure 1:**
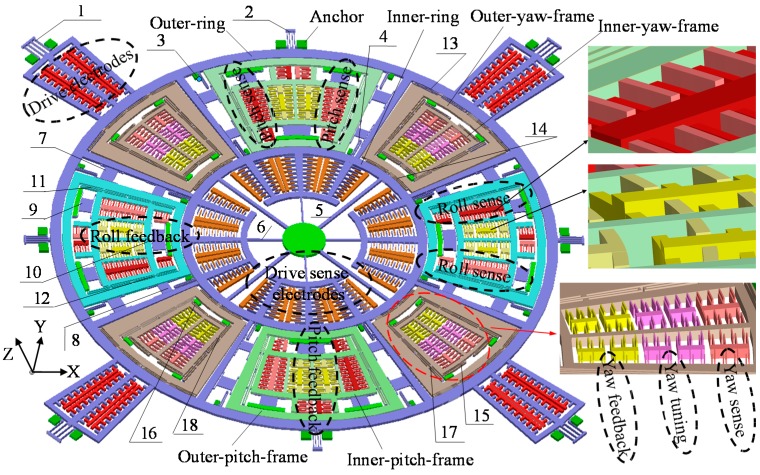
Schematic diagram of the triaxial vibratory wheel gyroscope.

Figure 1 shows the 3D schematic diagram of the designed wheel type triaxial gyroscope. It is a perfectly symmetric structure consisting of an outer-ring, an inner-ring, four yaw frames, four pitch/roll frames, four drive beams and four drive sense beams. The outer-ring and inner-ring are connected with each other by eight sticks, leaving eight regions to place the sensing frames. The drive beams are distributed at the outside of the outer-ring, while the drive sense frames are arranged inside the inner-ring.

In this design, several types of arc springs are described in this case to decouple drive and other sense modes, including the double folded spring Nos. 1 and 2, the U-shaped spring Nos. 3, 4, 15 and 16, straight spring Nos. 5 and 6, out-of-plane decoupling spring Nos. 7, 8, 9 and 10, double-U-shaped spring Nos. 11, 12, 13, 14, 17 and 18. The spring description are summarized in Table 1. Thanks to the elaborately designed springs, the drive beam, inner-yaw-frame and inner-pitch/roll-frame have only 1-degree of freedom (DOF) in their corresponding working directions respectively, which means that these modes can be fully decoupled effectively. The working principle of the drive and sense modes of the triaxial gyroscope will be introduced is the following section.

**Table 1:** Description of the springs.

Spring Number	Description	Connected with	DOF
1, 2	Double folded spring	Outer-ring	1-DOF around *z*-axis
3, 4	U-shaped spring	Outer/inner-ring	
5, 6	Straight spring	Inner-ring	
7, 8, 9, 10	Out-of-plane decoupling spring	Pitch/roll frame	1-DOF along *z*-axis
11, 12	Double-U-shaped spring		1-DOF around *z*-axis
13, 14	Double-U-shaped spring	Yaw frame	1-DOF along yaw sense direction
15, 16	U-shaped spring		
17, 18	Double-U-shaped spring		1-DOF around *z*-axis

#### 2.1.1. The Drive Mode

This device is made to be synchronously driven by the comb driving fingers arranged at the circumference of the outer-ring. When applying a certain electrostatic force by the comb drive electrodes, the whole wheel frame, including the outer-ring, outer-yaw-frame, outer-pitch/roll-frame and inner-ring are driven to rotate around *z*-axis. Also, the inside yaw-sense frame and the inside pitch/roll-sense frames cannot be dragged by the applied electrostatic force since the relevant springs (such as spring Nos. 9, 10, 15 and 16) bonded on the anchors possess considerable stiffness for the drive axis. Thus the couplings from drive to different sense modes are minimized to an extreme.

#### 2.1.2. The Yaw Mode

Once a rotation about the *z*-axis happens, an in-plane radial motion perpendicular with the drive direction will be induced for the outside yaw frame under the Coriolis effect. Correspondingly, the inside yaw frame should make a linkage motion with the outside yaw frame in the same radial orientation, so that the angular rate is converted to the varied capacitance value of the yaw sense parallel plates. Thus the outside yaw frame has 2-DOF in the drive and sense direction, while the inside yaw frame has 1-DOF in the sense orientation. Four yaw sense frames are evenly located between the outer-ring and inner-ring. To improve the yaw sense performance, the *z*-axis rotation is specified to be measured differentially using interdigital electrodes at each inside yaw frame. In addition, some frequency trimming electrodes for mode matching and feedback electrodes for the closed-loop working mode are also arranged in the inside yaw frame respectively.

#### 2.1.3. The Pitch/Roll Mode

As shown in Figure 1, the *x*-axis rotation rate is differentially measured by double symmetrical roll frames. When there occurs a spin about the *x*-axis exerted on the gyroscope, the outer-roll-frame will generate an out-of-plane translational motion in the *z*-axis under the Coriolis effect. Thus, the inner-roll-frame will be pulled to undergo a synchronized motion with the outer-roll-frame in the direction of the roll sense. Thus the outer-roll-frame has 2-DOF in the drive and sense direction, while the inner-roll-frame has 1-DOF in the sense direction. Furthermore, the roll mode comb fingers are etched with uneven thickness in the *z*-axis, which ensures that the *z*-axis motion capacitive detection is proportional to the Coriolis force. In this work, the key highlight for the structure is embodied in the decoupling mechanism including the in-plane motion and the out-of-plane motion in the drive and pitch and roll modes. Therefore, the relatively thin out-of-plane springs Nos. 7, 8, 9 and 10 compared to the regulated device are given. Because a relatively small stiffness in the *z*-axis direction and a very big stiffness in the lateral axis direction are arranged, they can be adopted similarly in pitch and roll modes to achieve the decoupling function from drive to sense modes. Besides, the frequency tuning parallel plate for mode matching is arranged under the outer-roll-frame. The feedback electrodes for the closed-loop working mode are placed in inner-roll frame.

The rotation velocity about the *y*-axis is differentially sensed by the pitch mode sense frames. The operation principle is very similar to that of roll mode.

### 2.2. Mathematical Model

To establish the mathematical model of the triaxial gyroscope, the whole system can be taken as a 4-DOF movement system, including a rotational movement around *z*-axis in drive mode, the linear in-plane movement in yaw sense mode and the rotational movement of the pitch/roll sense frames around the *x*/*y*-axes in pitch and roll modes. Under this condition, Lagrangian theory can be utilized to explain this system [18]. Some generalized coordinates will be chosen as *θ*, *α*, *β* and *y*, where *θ* denotes rotational angle in the drive mode structure; *α* is the rotational angle of the pitch-sense frame around the *x*-axis; *β* is the rotational angle of the roll sense frame around the *y*-axis; *y* is the translational motion of the yaw sense frame in the yaw sense direction. The 4-DOF dynamical Lagrangian functions can be written as:

(1) $$\begin{array}{l} L=T_{1}+4T_{2}+4T_{3}+2T_{4}+2T_{5}+2T_{6}+2T_{7}-4U_{1}-4U_{2}-8U_{3}-\\ 8U_{4}-4U_{5}-4U_{6}-4U_{7}-4U_{8}-4U_{9}-2U_{10}-4U_{11}-4U_{12}-\\ 8U_{13}-4U_{14}-8U_{15}-8U_{16}-8U_{17}-8U_{18} \end{array}$$

where *T*_1_ is the kinetic energy of the outer-ring and inner-ring; *T*_2_ and *T*_3_ are the kinetic energies of the outer-pitch frame, and inner-pitch frame respectively; *T*_4_ and *T*_5_ are the kinetic energies of the outer-roll-frame and inner-roll-frame, respectively; *T*_6_ and *T*_7_ are kinetic energies of the outer-yaw-frame and inner-yaw-frame respectively; *U_i_* (*i* =1, 2, …, 18) are the elastic potential energies of the springs shown in Figure 1, respectively; the subscripts of refer to each of the springs, respectively. Assuming that the input angular rates around *x*, *y*, *z*-axes are Ω*x*, Ω*y* and Ω*z* and the kinetic energies and elastic potential energies correspond to the number of the corresponding sense frames, respectively, then the kinetic energy of the outer-ring and inner-ring can be expressed as:

(2) $$T_{1}=\frac{1}{2}J_{1x}{\text{Ω}}_{x}^{2}+\frac{1}{2}J_{1y}{\text{Ω}}_{y}^{2}+\frac{1}{2}J_{1z}{\left({\text{Ω}}_{z}+\dot{\text{θ}}\right)}^{2}$$

where *J_1x_*, *J_1y_*, *J_1z_* are the inertia moment with the outer-ring and inner-ring about *x*, *y*, *z*-axes respectively.

The kinetic energies of the outer-yaw-frame and inner-yaw-frame are:

(3) $$T_{2}=\frac{1}{2}J_{2x}{\text{Ω}}_{x}^{2}+\frac{1}{2}J_{2y}{\text{Ω}}_{y}^{2}+\frac{1}{2}J_{2z}{\left({\text{Ω}}_{z}+\dot{\text{θ}}\right)}^{2}+\frac{1}{2}m_{y1}{\dot{y}}^{2}$$

(4) $$T_{3}=\frac{1}{2}J_{3x}{\text{Ω}}_{x}^{2}+\frac{1}{2}J_{3y}{\text{Ω}}_{y}^{2}+\frac{1}{2}J_{3z}{\text{Ω}}_{z}^{2}+\frac{1}{2}m_{y2}{\dot{y}}^{2}$$

where *J*_2*x*_, *J*_2*y*_, *J*_2*z*_ are the moment of inertia of the outer-yaw-frame around the *x*, *y*, *z*-axis, respectively; *J*_3*x*_, *J*_3*y*_, *J*_3*z*_ are the moment of inertia of the inner-yaw-frame around the *x*, *y*, *z*-axes, respectively; *m_y_*_1_, *m_y_*_2_ are the masses of the two frames respectively.

Similarly, the kinetic energies of the outer-pitch-frame and inner-pitch-frame are:

(5) $$T_{4}=\frac{1}{2}J_{4x}{\left({\text{Ω}}_{x}+\dot{\text{α}}\right)}^{2}+\frac{1}{2}J_{4y}{\text{Ω}}_{y}^{2}+\frac{1}{2}J_{4z}{\left({\text{Ω}}_{z}+\dot{\text{θ}}\right)}^{2}$$

(6) $$T_{5}=\frac{1}{2}J_{5x}{\left({\text{Ω}}_{x}+\dot{\text{α}}\right)}^{2}+\frac{1}{2}J_{5y}{\text{Ω}}_{y}^{2}+\frac{1}{2}J_{5z}{\text{Ω}}_{z}^{2}$$

where *J*_4*x*_, *J*_4*y*_, *J*_4*z*_ are the moment of inertia of the outer-pitch-frame around the *x*, *y*, *z*-axes, respectively; *J*_5*x*_, *J*_5*y*_, *J*_5*z*_ are the moment of inertia of the inner-pitch-frame around the *x*, *y*, *z*-axes, respectively.

Due to the structural symmetry, the moments of inertia of the roll frame around *x*, *y*, *z*-axes are the same with that of the pitch frame around *x*, *y*, *z*-axes respectively. Thus the kinetic energies of the outer-roll-frame and inner-roll-frame are:

(7) $$T_{6}=\frac{1}{2}J_{4y}{\text{Ω}}_{x}^{2}+\frac{1}{2}J_{4x}{\left({\text{Ω}}_{y}+\dot{\text{β}}\right)}^{2}+\frac{1}{2}J_{4z}{\left({\text{Ω}}_{z}+\dot{\text{θ}}\right)}^{2}$$

(8) $$T_{7}=\frac{1}{2}J_{5y}{\text{Ω}}_{x}^{2}+\frac{1}{2}J_{5x}{\left({\text{Ω}}_{y}+\dot{\text{β}}\right)}^{2}+\frac{1}{2}J_{5z}{\text{Ω}}_{z}^{2}$$

Since the rotational movements in the drive and sense modes are within small ranges (typically with a rotation angle less than 1°), the displacement of a spring can be approximately calculated by multiplying its corresponding radius and rotation angle. Thus the elastic potential energies of the springs can be expressed as:

(9) $$U_{i}=\left\{ \begin{array}{l} \frac{1}{2}k_{i}{\left(R_{i}\text{θ}\right)}^{2},\begin{array}{cc} & i=1,2,\cdots ,6,11,12,17,18 \end{array}\\ \frac{1}{2}k_{i}{\left(R_{i}\text{α}\right)}^{2}+\frac{1}{2}k_{i}{\left(R_{i}\text{β}\right)}^{2},\begin{array}{cc} & i=7,8,9,10 \end{array}\\ \frac{1}{2}k_{i}y^{2},\begin{array}{cc} & i=13,14,15,16 \end{array} \end{array}\right.$$

where *k_i_* (*i* = 1, 2, …, 18) are the stiffnesses of the springs along their motion directions, respectively; *R_i_* (*i* = 1, 2, …, 12, 17, 18) are the equivalent radii of the corresponding springs, respectively.

Taking the energy dissipation caused by the damping into consideration, the dissipated energy can be expressed as:

(10) $$D=\frac{1}{2}c_{1}{\dot{\text{θ}}}^{2}+\frac{1}{2}c_{2}({\dot{\text{α}}}^{2}+{\dot{\text{β}}}^{2})+\frac{1}{2}c_{3}{\dot{y}}^{2}$$

where *c*_1_, *c*_2_, *c*_3_ are the damping coefficients of the moving structures in the drive, pitch/roll and yaw modes along their motion directions, respectively.

Thus the dynamic equations of the tri-axis gyroscope can be deduced by the Lanrange function as:

(11) $$\left\{ \begin{array}{l} \frac{d}{dt}\left(\frac{\partial L}{\partial \dot{\text{θ}}}\right)-\frac{\partial L}{\partial \text{θ}}+\frac{\partial D}{\partial \dot{\text{θ}}}=M_{d},\begin{array}{ccc} & & \frac{d}{dt}\left(\frac{\partial L}{\partial \dot{\text{α}}}\right)-\frac{\partial L}{\partial \text{α}}+\frac{\partial D}{\partial \dot{\text{α}}}=M_{a} \end{array}\\ \frac{d}{dt}\left(\frac{\partial L}{\partial \dot{\text{β}}}\right)-\frac{\partial L}{\partial \text{β}}+\frac{\partial D}{\partial \dot{\text{β}}}=M_{b},\frac{d}{dt}\left(\frac{\partial L}{\partial \dot{y}}\right)-\frac{\partial L}{\partial y}+\frac{\partial D}{\partial \dot{y}}=F_{y}\cos\text{ϕ},\begin{array}{c} \begin{array}{cc} & \text{ϕ}\in \left[0,\begin{array}{c} \text{θ} \end{array}\right] \end{array} \end{array} \end{array}\right.$$

where *M_d_* is the moment in the drive mode; *M_a_*, *M_b_* are the Coriolis moments in the pitch and roll modes along *x*, *y*-axes, respectively; *F_y_* is the Coriolis force in the yaw mode; ϕ is the angle between the real the yaw sense direction and the orientation of Coriolis force in the yaw mode.

Substituting Equations (1)–(10) into Equation (11), the motion equations of the tri-axis gyroscope can be obtained as:

(12) $$\begin{array}{l} (2J_{1z}+4J_{2z}+4J_{4z})\ddot{\text{θ}}+c_{1}\dot{\text{θ}}+4(\sum_{i=1,2,5,6,11,12}k_{i}R_{i}+2\sum_{j=3,4,17,18}k_{j}R_{j})\text{θ}\\ =M_{d}-(2J_{1z}+4J_{2z}+4J_{4z}){\dot{\text{Ω}}}_{z} \end{array}$$

(13) $$2(J_{4x}+J_{5x})\ddot{\text{α}}+c_{2}\dot{\text{α}}+2(2\sum_{i=7,8,9}k_{i}R_{i}+k_{10}R_{10})\text{θ}=M_{a}-2(J_{4x}+J_{5x}){\dot{\text{Ω}}}_{x}$$

(14) $$2(J_{4x}+J_{5x})\ddot{\text{β}}+c_{2}\dot{\text{β}}+2(2\sum_{i=7,8,9}k_{i}R_{i}+k_{10}R_{10})\text{β}=M_{b}-2(J_{4x}+J_{5x}){\dot{\text{Ω}}}_{y}$$

(15) $$4(m_{y1}+m_{y2})\ddot{y}+c_{3}\dot{y}+4(2k_{13}+k_{14}+2k_{15}+2k_{16})y=F_{y}\cos\text{ϕ}$$

From the dynamic equations of the gyroscope shown in Equations (11)–(15), the resonant frequencies in the drive and sense modes can be easily expressed as:

(16) $$f_{d}=\frac{1}{2\pi }\sqrt{\frac{2(\sum_{i=1,2,5,6,11,12}k_{i}R_{i}+2\sum_{j=3,4,17,18}k_{j}R_{j})}{\left(J_{1z}+2J_{2z}+2J_{4z}\right)}}$$

(17) $$f_{p}=f_{r}=\frac{1}{2\pi }\sqrt{\frac{2\sum_{i=7,8,9}k_{i}R_{i}+k_{10}R_{10}}{J_{4x}+J_{5x}}}$$

(18) $$f_{y}=\frac{1}{2\pi }\sqrt{\frac{2k_{13}+k_{14}+2k_{15}+2k_{16}}{m_{y1}+m_{y2}}}$$

where *f_d_*, *f_y_*, *f_p_* and *f_r_* are the corresponding resonance frequencies for drive, yaw, pitch and roll modes.

### 2.3. Structural Dimensions Design

According to the previous schematic diagram of this device, the whole size including the springs can be calculated by the assumed driving force, scale factors and equivalent qualities in these modes. Therefore, the proposed triaxial gyroscope with fully decoupled springs with certain drive force and coincident sensitivity in different sense modes can be figured out. The specific sizes are summarized in Table 2.

**Table 2:** Part of the structure dimensions.

Parameters	Values
Total die size	9800 μm × 9800 μm
Structure thickness (*h*)	60 μm
Drive mode	
Drive comb length and overlap length	48 μm, 24 μm (average)
Drive comb width and gap	5 μm, 3 μm
Drive comb number	1212
Drive-sense static capacitance	6.25 pF
Moment of inertia of two rings (*J*_1*z*_)	3.51 × 10^−11^ kg·m^2^
Moment of inertia of outer-yaw-frame (*J*_2*z*_)	2.56 × 10^−12^ kg·m^2^
Moment of inertia of outer-pitch/roll-frame (*J*_4*z*_)	2.81 × 10^−12^ kg·m^2^
Yaw mode	
Parallel plate length and overlap length	120 μm, 100 μm (average)
Parallel plate width and gaps (*w*, *d*_1_ and *d*_2_)	5 μm, 3 μm and 15 μm
Yaw sense static capacitance	0.74 pF × 4
Feedback static capacitance	0.41 pF
Stiffness tuning static capacitance	0.52 pF
Masses of outer/inner-yaw-frames (*m_y_*_1,_*m_y_*_2_)	0.213 mg, 0.082 mg
Pitch/roll mode	
Comb finger length and overlap length	100 μm, 85 μm (average)
Comb finger width and gap	5 μm, 3 μm
Comb finger thickness and overlap thickness	45 μm, 30 μm
Out-of-plane decoupling spring thickness	30 μm
Pitch/roll sense capacitance	1.27 pF × 2
Feedback static capacitance	0.32 pF
Stiffness tuning parallel plate area and gap (*S*’, *d*_3_)	1.47 × 10^−6^ m^2^, 3 μm
Moment of inertia of outer-pitch/roll-frame (*J*_4*x*_)	1.76 × 10^−12^ kg·m^2^
Moment of inertia of inner-pitch/roll-frame (*J*_5*x*_)	1.59 × 10^−12^ kg·m^2^

## 3. Mode Matching

### 3.1. Spring Design

As known to all, the mode matching between the drive and other sense modes will greatly enhance the different sense axis quality factor to further increase sensitivity, SNR and relatively matched readout circuit with a device [19]. The frequency split between drive mode and other sense modes should be decreased to improve the performance of the triaxial gyroscope. Therefore, after defining the frames and rings dimensions of different modes, the optimized algorithm will be further investigated for efficient mode matching.

Since the basic motion curve of the vibratory wheel gyroscope is arc-shaped, part of the springs are designed to be arcuate sides, so that the springs are in accordance with the movement of the structure. To find out the influence of arcuate sides on the stiffnesses of the springs, the FEA software ANSYS is used to analyze the stiffnesses of all the springs shown in Figure 1. The different spring stiffness calculations can be summarized in Table 3 [5,20], where the Young’s modulus *E* is 1.86 × 10^11^ Pa; the shear modulus *G* is 5.75 × 10^10^ Pa; coefficient *λ* is 0.089; the decoupled spring thickness in out-of-plane *h_p_* is 30 μm.

**Table 3:** The stiffness expressions of different springs.

Springs	Dimensions	Stiffness Expressions
Double folded springs: 1, 2	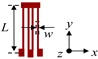	Stiffness in *x*-axis: $k_{x}=Eh{\left(w/L\right)}^{3}$
U-shaped springs: 3, 4	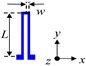	Stiffness in *x*-axis: $k_{x}=Eh{\left(w/L\right)}^{3}/2$
Straight springs: 5, 6	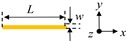	Stiffness in *y*-axis: $k_{y}=Eh{\left(w/L\right)}^{3}$
Out-of-plane decoupling springs: 7, 8	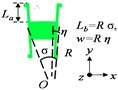	Stiffness in *z*-axis: $k_{z}=\frac{16\text{λ}Gh_{p}w^{3}}{L_{a}L_{b}^{2}}$, simulated errors are less than 0.4%.
Out-of-plane decoupling springs: 9, 10	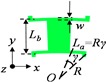	
Double-U-shaped spring: 11, 12, 17, 18	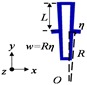	Stiffness in *x*/*y*-axis: $k=Eh{\left(w/L\right)}^{3}$, simulated errors are less than 0.2%.
Double-U-shaped springs: 13, 14	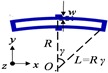	
U-shaped springs: 15, 16	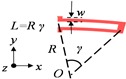	Stiffness in *y*-axis: $k=Eh{\left(w/L\right)}^{3}/2$, simulated error is less than 0.2%.

### 3.2. AFSA for Mode Matching

To finalize the matching among the drive and sense modes, the springs sizes in each mode should be elaborately designed. Traditionally, a single-axis gyroscope mode matched procedure was achieved through iteratively trimming the relevant spring sizes and simulating the matched results in a FEM tool. Unfortunately, in this design the springs necessary for this kind of triaxial gyroscope are so numerous, that it would be quite time consuming if the traditional mode match processing is adopted. Actually, our task has become a multiple parameters optimization problem so far. In the past several decades, a lot of intelligence optimization algorithms have been developed and successfully used in many fields of application, where swarm intelligence (SI) optimization is a certain class of population-based metaheuristics which are inspired by the behavior of swarm of local agents interaction with each other and the environment. SI is relatively new subfield of artificial intelligence. The classical SI algorithms include PSO, artificial bee colony (ABC), ant colony optimization (ACO) and artificial fish swarm algorithm (AFS), *etc.* For simplification and feasibility, due to the fact our focus is on the whole structure design process, and not the running through all the SI algorithms above here, we choose the basic PSO and AFS for comparison here, especially the PSO which has been used in our previous research.

As for these novel nonlinear algorithms, there are still no systematic or complete mathematical proofs to verify and compare them. However, they are widely and affirmatively used. From experience, PSO has the characteristics of a merely local convergence rate, high efficiency and sensitivity to the initial value and parameter selection. In this case, the AFS will be chosen for its increased simplicity, global optimization ability, and fast tracking over the objective function parameters drift. Meanwhile, the feasible evaluation criteria have been figured out to assess the results derived by the SI algorithm for specific applications [21]. In this work, a solving assessment approach to SI optimization is presented by experimental analysis according to searching range analysis and its characteristics. The feasible evaluation criteria were regulated based on the distance to divide solution samples into several parts using solving space, “good enough” sets and relevant statistics knowledge. For the verification of different approaches, some typical intelligent algorithms are taken into comparison. We can find that, in their opinion, PSO and AFS are both excellent in obtaining the optimal solution, however AFS reflects its obvious advantage in a stable accuracy solution despite of the swarm size and the number of iteration.

The artificial fish swarm algorithm is one the best optimization algorithms based on the natural swarm behavior of the collective movement of the fish. It is an intelligent algorithm which can be used for many applications such as optimizing, image processing, controlling, data mining, *etc.* [22,23]. It possesses the advantages of fast convergence speed, flexibility, fault tolerance and high accuracy. The algorithm starts with a group of random initial solutions abstracted as fishes. Then each fish performs a search to approach the global optimum result interactively in the solution space. The algorithm terminates while the maximum number of iterations exceeds a certain value, or while the fitness value can meet the requirement of accuracy. In the design of a mode-matched triaxial gyroscope using the AFSA, the objective function can be chosen as a relevant equation regarding the resonant frequencies for all drive and sense modes

(19) $$F={\left(f_{d}-f_{obj}\right)}^{2}+{\left(f_{y}-f_{obj}\right)}^{2}+{\left(f_{p}-f_{obj}\right)}^{2}$$

where; *f_obj_* = 8 kHz is the expected frequency for all drive and sense modes.

Since the structure dimensions have been determined, the equivalent radii of the springs *R_i_* (*i =* 1, 2, …, 18) can be easily obtained. By substituting Equations (11)–(15) into Equation (19), the object function is taken as a function of the springs’ stiffness. To reduce the number of dependent variables and simplify the optimization process, the variables are classified as:

(20) $$x(i)=\left\{ \begin{array}{l} \frac{w_{i}}{L_{i}},\begin{array}{cc} & i=1,2,\cdots ,6,11,12\cdots ,18 \end{array}\\ \begin{array}{cc} \frac{w_{i}^{3}}{L_{ia}L_{ib}^{2}}, & i=7,8,9,10 \end{array} \end{array}\right.$$

According to the stiffness expressions of different springs summarized in Table 3 and the expressions of resonant frequencies in Equations (11)–(15), the objective function can be further expressed by variables *x*(*i*) as:

(21) $$\begin{array}{l} F={\left[\frac{1}{2\pi }\sqrt{\frac{2Eh(\sum_{i=1,2,5,6,11,12}x^{3}(i)R_{i}+\sum_{j=3,4}x^{3}(j)R_{j}+2\sum_{m=17,18}x^{3}(m)R_{m})}{J_{1z}+2J_{2z}+2J_{4z}}}-f_{obj}\right]}^{2}+\\ \begin{array}{cc} & {\left[\frac{1}{2\pi }\sqrt{\frac{16\text{λ}Gh_{p}(\sum_{i=7,8,9}x(i)R_{i}+x(10)R_{10})}{J_{4x}+J_{5x}}}-f_{obj}\right]}^{2} \end{array}+{\left[\frac{1}{2\pi }\sqrt{\frac{2Ehx^{3}(13)+Eh\sum_{i=14,15,16}x^{3}(i)}{m_{y1}+m_{y2}}}-f_{obj}\right]}^{2} \end{array}$$

Because the spaces for placing the springs are limited by the frames, the ranges of changing spring dimensions can be chosen by the structural restrictions and experiences as:

(22) $$x(i)\in \left\{ \begin{array}{l} \left[10/550,15/300\right],\begin{array}{cc} & i=1,2,3,4;\begin{array}{ccc} & & \left[15/600,40/600\right],\begin{array}{cc} & i=5 \end{array} \end{array} \end{array}\\ \left[40/1500,80/1500\right],\begin{array}{cc} & i=6;\begin{array}{ccc} & & \left[6^{3}/180/320^{2},10^{3}/60/320^{2}\right],\begin{array}{cc} & i=7 \end{array} \end{array} \end{array}\\ \left[6^{3}/180/235^{2},10^{3}/60/235^{2}\right],\begin{array}{cc} & i=8,9,10 \end{array}\\ \left[9/450,15/350\right]\begin{array}{cc} & i=11,12,17,18; \end{array}\begin{array}{ccc} & & \left[9/550,15/350\right]\begin{array}{cc} & i=13,14,15,16 \end{array} \end{array} \end{array}\right.$$

Then the mode-match procedure of the triaxial gyroscope by AFSA will be separated into some steps:

*Step 1: Parameter Initialization*. The adjusting ranges of the parameters are started from Equation (22); then a swarm of fishes with *Size* = 100 is randomly selected in the solution space, where *X_i_* denotes the position of the fishes numbered *i* = 1, 2, …, *Size*. The parameters are initialized as shown in Table 4.

**Table 4:** Parameters of AFSA.

Parameters	Values	Parameters	Values
Swarm of fishes (*Size*)	100	Visual distance (*Vis*)	1
Maximum evolution generations (*Gen*)	200	Maximum try number in preying (*try_num*)	100
Moving step (*step*)	0.1	Congesting factor (*delta*)	0.618

*Step 2: AF_Prey*. In the visual distance of current state *X_i_*, a state *X_j_* is randomly selected as:

(23) $$X_{j}=X_{i}+rand()\cdot Vis,n=n+1$$

Then the next state can be expressed as:

(24) $$X_{i\_next}=\left\{ \begin{array}{l} X_{i}+rand()\cdot step\cdot (X_{j}-X_{i})/\left\|X_{j}-X_{i}\right\|,\begin{array}{cc} & F(X_{i})>F(X_{j}) \end{array}\\ returnbacktoequation(34),\begin{array}{cc} F(X_{i})<F(X_{j}), & n<try\_num \end{array}\\ X_{i}+rand()\cdot Vis,\begin{array}{cc} & F(X_{i})<F(X_{j}), \end{array}n<try\_num \end{array}\right.$$

*Step 3: AF_Swarm*. In the visual region of current state *X_i_*, *X_c_* is the center position and *n_f_* is the number of its companions. The next state can be expressed as:

(25) $$X_{i\_next}=\left\{ \begin{array}{l} X_{i}+rand()\cdot step\cdot (X_{c}-X_{i})/\left\|X_{c}-X_{i}\right\|,F(X_{c})/n_{f}<delta\cdot F(X_{i})\\ Step2,\begin{array}{ccc} & & \begin{array}{cc} & F(X_{c})/n_{f}>delta\cdot F(X_{i}) \end{array} \end{array} \end{array}\right.$$

*Step 4: AF_ Follow*. Like Step 3, *X_j_* is the position with the maximum value in the visual region of current state *X_i_*, and *n_f_* is the number of its companions. Then the next state can be expressed as:

(26) $$X_{i\_next}=\left\{ \begin{array}{l} X_{i}+rand()\cdot step\cdot (X_{j}-X_{i})/\left\|X_{j}-X_{i}\right\|,F(X_{j})/n_{f}<delta\cdot F(X_{i})\\ Step2,\begin{array}{ccc} & & \begin{array}{cc} & F(X_{j})/n_{f}>delta\cdot F(X_{i}) \end{array} \end{array} \end{array}\right.$$

*Step 5: Check the end condition, and return to Step 3 or end the optimization process*. Finally, the optimized *X_i_* is obtained which has the minimum objective function value in the problem space.

**Figure 2:**
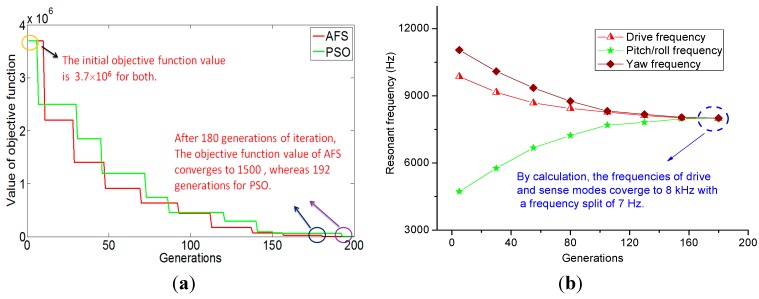
Results of the AFSA algorithm. (**a**) Objective function value *versus* generations; (**b**) Resonant frequencies obtained by calculation *versus* generations.

After the discussions above, the AFSA algorithm simulation will be further performed in Matlab. Figure 2 plots the final results. In this case, to compare the performance between the PSO and AFS SI algorithms, as shown in Table 5, both algorithms are conducted using the same maximum number of evolution steps (200). Under the same initial values and swarm size, PSO can converge to the optimal value with an error of 0.1% after at least 192 generations while it takes 180 generations for AFS, which obviously tells us that AFS is relatively faster. Besides, for the final accuracy value, AFS will be better than PSO. Their objective function value versus generations is depicted in Figure 2a. In Figure 2b, the drive and different sense modal frequencies are derived by Equations (16)–(18). According to the optimization data and our experiences on the previous design, Table 6 lists all the necessary optimized spring parameters.

**Table 5:** The comparison between PSO and AFS SI algorithms.

SI Algorithm	Needed Evolution Generations	Swarm Size	Objective Function Error	Frequency Split
PSO	>192	100	<0.1%	150 Hz
AFS	>180	100	<0.1%	108 Hz

**Table 6:** The optimized values of the variables and springs dimensions.

**Variables**	***x*(1)**	***x*(2)**	***x*(3)**	***x*(4)**	***x*(5)**	***x*(6)**	***x*(7)**	***x*(8)**	***x*(9)**
**Values** (μm/μm)	0.0259	0.0254	0.0262	0.0248	0.0473	0.0407	4.098 × 10^−5^	2.475 × 10^−5^	4.007 × 10^−5^
**Dimensions** (μm/μm)	$\frac{10}{386}$	$\frac{10}{394}$	$\frac{10}{382}$	$\frac{10}{403}$	$\frac{28.4}{600}$	$\frac{61}{1500}$	$\frac{8^{3}/122}{320^{2}}$	$\frac{6^{3}/158}{235^{2}}$	$\frac{7^{3}/155}{235^{2}}$
**Variables**	***x*(10**)	***x*(11)**	***x*(12)**	***x*(13)**	***x*(14)**	***x*(15)**	***x*(16)**	***x*(17)**	***x*(18)**
**Values** (μm/μm)	4.374 × 10^−5^	0.0267	0.0213	0.0182	0.0190	0.0173	0.0220	0.0303	0.0241
**Dimensions** (μm/μm)	$\frac{7^{3}/142}{235^{2}}$	$\frac{11.2}{420}$	$\frac{8.8}{420}$	$\frac{9.3}{510}$	..	$\frac{9}{520}$	$\frac{8.8}{400}$	$\frac{11.8}{390}$	$\frac{9.4}{390}$

To validate the efficiency of AFSA, the obtained spring parameters are input into the modeling in ANSYS. The matching simulation results tell that the resonant frequencies for drive, yaw and pitch/roll modes are 7924, 8010 and 8032 Hz, respectively. The frequency split is 108 Hz, which is greater than the computation result in Figure 2b. It can be strated in this case that the spring stiffness computation equations through AFSA cannot achieve the desired solution.

### 3.3. Further Mode Matching by Experience

**Figure 3:**
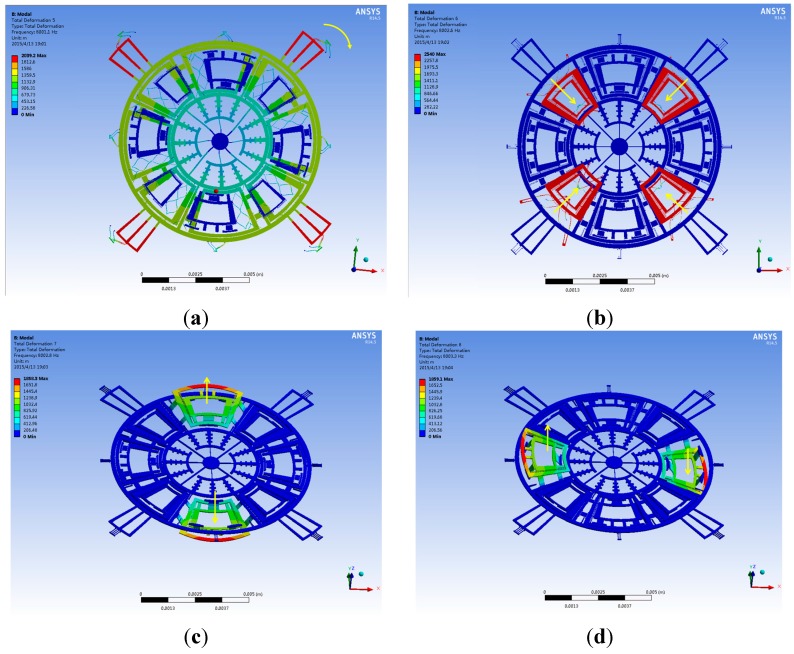
The desired modes of the tri-axis gyroscope: (**a**) The drive mode; (**b**) The yaw mode; (**c**) The pitch mode; (**d**) The roll mode.

**Figure 4:**
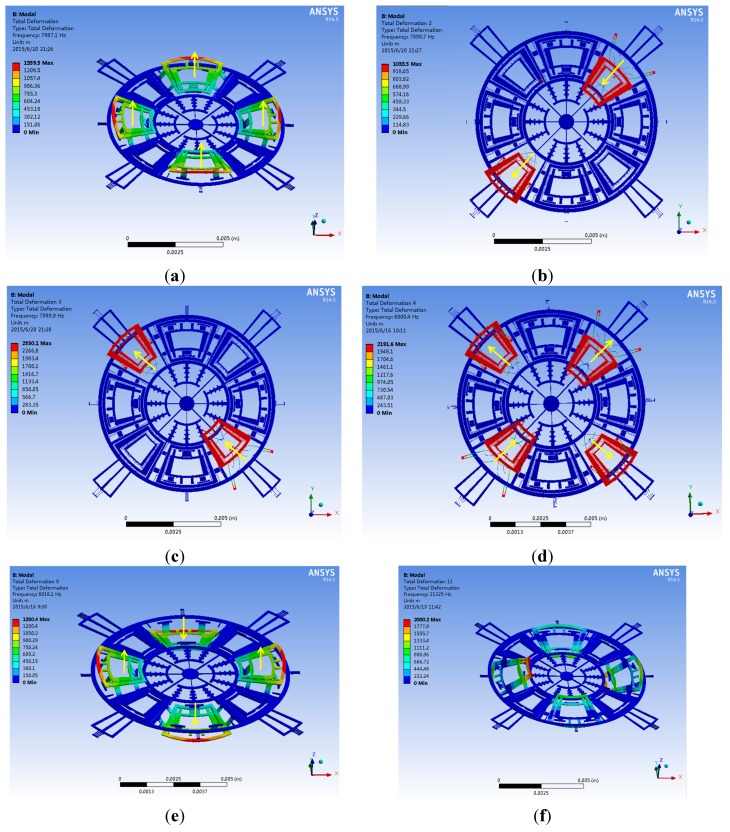
The interference modes of the tri-axis gyroscope: (**a**,**e**,**f**) The interference modes of pitch/roll frames; (**b**–**d**) The interference modes of yaw frames.

By AFSA, the drive and sense modes resonant frequencies have been matched around 8 kHz with a split of 108 Hz. Since a higher matching is necessary in the drive and other sense modes, the corresponding spring dimensions will be slightly trimmed by experience. According to the stiffness expressions and motion directions of different springs summarized in Table 3, the post adjustment of the springs’ dimensions can be distributed into three parts. The first part is to adjust the drive mode frequency by changing the lengths of spring Nos. 1, 2, 3. The second part is the adjustment of the yaw mode frequency by changing the lengths of spring No. 14. The third part is to adjust the pitch/roll mode frequency by changing the lengths of spring Nos. 7, 8. The post adjustment results are summarized in Table 7. After four steps of trimming of the springs’ parameters, the drive, yaw, pitch and roll modes resonant frequencies will further reach 8001.1, 8002.6, 8002.8 and 8003.3 Hz respectively. The detailed modal matching results are displayed in Figure 3 and Figure 4 and Table 8. Obviously, a small split of 2.2 Hz is achieved after the mode matching post process.

The bandwidth and sensitivity are contradict with each other for a certain gyroscope structure, a trade-off should be made in a carefully designed gyroscope. The bandwidth of a gyroscope can be approximately calculated by its frequency split, while the quality factor can be expressed by the quotient of resonant and frequency split. If the triaxial gyroscope is designed as using the AFSA obtained results, the bandwidth is 108 Hz. After further mode matching, the quality factor is dramatically enhanced and the bandwidth decreased to 2.2 Hz. To improve the bandwidth in this case, the triaxial gyroscope can be designed to work in the closed-loop sense modes [4].

**Table 7:** The changed springs dimensions for further mode matching.

Steps	*x*(1)	*x*(2)	*x*(3)	*x*(7)	*x*(8)	*x*(14)	Frequencies and Split in Three Modes (Hz)			
							Drive	Yaw	Pitch/Roll	Split
**Step 0 (initial)**	$\frac{10}{386}$	$\frac{10}{394}$	$\frac{10}{382}$	$\frac{8^{3}/122}{320^{2}}$	$\frac{6^{3}/158}{235^{2}}$	** $\frac{9.1}{480}$	7924	8010	8032	108
**Step 1**	$\frac{10}{380}$	/	/	$\frac{8^{3}/123}{320^{2}}$	/	$\frac{9.1}{485}$	7968	8008	8011	43
**Step 2**	/	$\frac{10}{390}$	/	$\frac{8^{3}/125}{320^{2}}$	/	$\frac{9.1}{486}$	7990.4	8005	8007	16.6
**Step 3**	/	/	$\frac{10}{380}$	/	$\frac{6^{3}/159}{235^{2}}$	$\frac{9.1}{487}$	8001.1	8003.4	8003.3	2.2
**Step 4 (last)**	$\frac{10}{380}$	$\frac{10}{390}$	$\frac{10}{380}$	$\frac{8^{3}/125}{320^{2}}$	$\frac{6^{3}/160}{235^{2}}$	$\frac{9.1}{487}$	8001.1	8002.6	8003.3	2.2

**Table 8:** Summary of the modal simulation results.

Mode	Frequency (Hz)	Description
1	7987.1	The interference mode: four pitch/roll frames in in-phase resonant mode
2	7999.7	The interference mode: two yaw frames in in-phase resonant mode
3	7999.9	The interference mode: another two yaw frames in in-phase resonant mode
4	8000.4	The interference mode: four yaw frames in anti-phase resonant mode
5	8001.1	The drive mode
6	8002.6	The yaw mode
7	8002.8	The pitch mode
8	8003.3	The roll mode
9	8018.1	The interference mode: four pitch/roll frames in in-phase resonant mode
10/11	11288/11325	The interference mode: four pitch/roll frames in rotational mode

## 4. Simulation Results and Fabrication Process

### 4.1. Frequency Tuning

It is not enough that the four modal frequencies are matched well only by simulation, as any fabrication imperfections will definitely cause extra frequencies splits. In this case, to eliminate this deviation induced by fabrication error, the assistant stiffness trimming approach need to be developed in this design. This kind of electronic trim technology can be borrowed from the [17,19].

#### 4.1.1. Yaw Mode Stiffness Trimming

**Figure 5:**
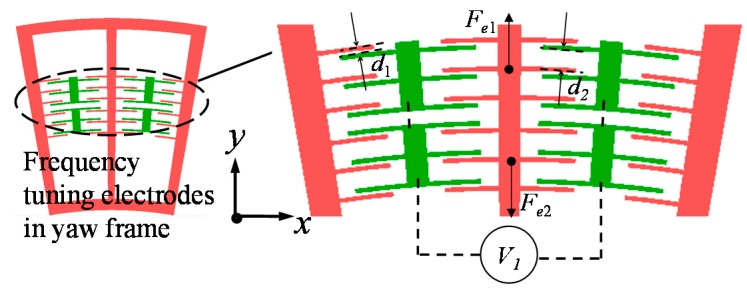
The frequency tuning electrodes in yaw mode.

Figure 1 shows the varying distance interdigital electrodes for yaw mode stiffness trimming, which are distributed evenly within the inner-yaw-frame. Its stiffness trimming mechanism is shown in Figure 5. For example, when the same DC voltage *V*_1_ is directly applied onto two groups of stiffness trimming electrodes, the two corresponding electrostatic forces are written as

(27) $$\left\{ \begin{array}{l} F_{e1}=\frac{N\varepsilon S}{2}\left[\frac{1}{{\left(d_{1}-\text{Δ}y\right)}^{2}}-\frac{1}{{\left(d_{2}+\text{Δ}y\right)}^{2}}\right]V_{1}^{2}\\ F_{e2}=\frac{N\varepsilon S}{2}\left[\frac{1}{{\left(d_{1}+\text{Δ}y\right)}^{2}}-\frac{1}{{\left(d_{2}-\text{Δ}y\right)}^{2}}\right]V_{1}^{2} \end{array}\right.$$

where *N* denotes one group electrodes number; *S* denotes the overlapping area between a pair of electrodes; *d*_1_ and *d*_2_ are the capacitive gaps; Δ*y* is the assumed small movement along the *y*-axis.

Then the resultant force generated by the frequency tuning electrodes is:

(28) $$\begin{array}{l} F_{e}=F_{e1}+F_{e2}=\frac{N\varepsilon SV_{1}^{2}}{2}\left[\frac{4d_{1}\text{Δ}y}{{\left(d_{1}-\text{Δ}y\right)}^{2}{\left(d_{1}+\text{Δ}y\right)}^{2}}+\frac{4d_{2}\text{Δ}y}{{\left(d_{2}-\text{Δ}y\right)}^{2}{\left(d_{2}+\text{Δ}y\right)}^{2}}\right]\\ \begin{array}{ccccc} & & & & \approx \end{array}2N\varepsilon SV_{1}^{2}(\frac{1}{{d_{1}}^{3}}+\frac{1}{{d_{2}}^{3}})\text{Δ}y \end{array}$$

Because *F_e_* can only generate the reversed force with its motion, the negative electrostatic stiffness should be:

(29) $$k_{e}=-\frac{F_{e}}{\text{Δ}y}=-2N\varepsilon SV_{1}^{2}(\frac{1}{{d_{1}}^{3}}+\frac{1}{{d_{2}}^{3}})$$

#### 4.1.2. Pitch/Roll Mode Stiffness Trimming

The stiffness trimming electrode plates will be placed beneath the pitch sense frame to sense an out-of-plane motion. Similarly, if a DC voltage *V*_2_ is directly applied onto the stiffness trimming electrode plates, its force is written as:

(30) $$F_{ez}=\frac{1}{2}\frac{\varepsilon S^{\prime }}{{\left(d_{3}-\text{Δ}z\right)}^{2}}V_{2}^{2}\approx \frac{1}{2}\frac{\varepsilon S^{\prime }}{{d_{3}}^{3}}(d_{3}+2\text{Δ}z)V_{2}^{2}=\underset{const}{\underset{︸}{\frac{1}{2}\frac{\varepsilon S^{\prime }}{{d_{3}}^{2}}V_{2}^{2}}}+\frac{\varepsilon S^{\prime }}{{d_{3}}^{3}}V_{2}^{2}\text{Δ}z$$

where *S*′ denotes the overlapping area between the electrode plate and the pitch sense frame; *d*_3_ denotes the capacitive gap along *z*-axis; Δ*z* denotes the pitch sense frame displacement along *z*-axis.

Similarly, the calculated *F_ez_* shows a reversed electrostatic force with its motion. Besides, a constant term exists in *F_ez_* expression, which does not depend on the trimmed stiffness. Therefore, the useful negative stiffness is expressed as:

(31) $$k_{ez}=-\frac{\varepsilon S^{\prime }}{{d_{3}}^{3}}V_{2}^{2}$$

Based on the negative stiffness Equations (29) and (31), it is clear that the sense mode frequencies can be tuned by DC voltages.

**Figure 6:**
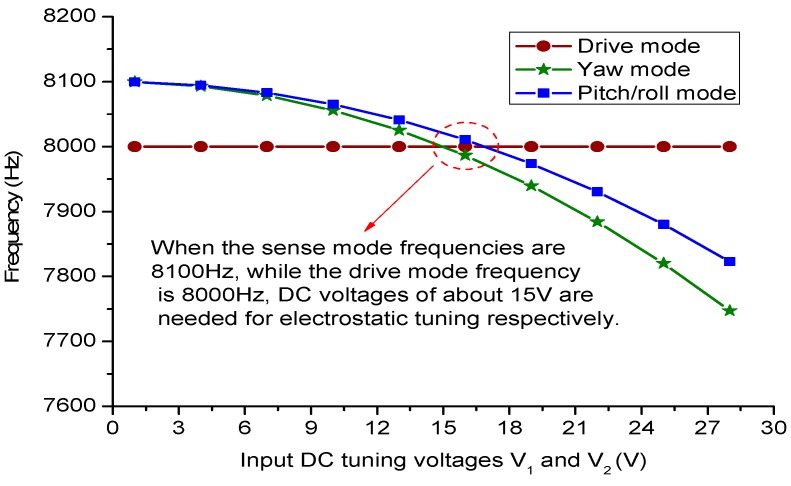
Electrostatic tuning simulation results of sense modes in ANSYS.

To explore relationships of tuned frequencies and input DC tuning voltages, the triaxial gyroscope are simulated in ANSYS modal analysis by setting the electromechanical transducer TRANS126. The TRANS126 element represents the capacitive response of the device to motion in one direction, thus it can simulate the process of electrostatic stiffness tuning. The obtained results are depicted in Figure 6.

### 4.2. Cross-Axis Effect Analysis

Though this device is originally conceived to eliminate several coupling effects among these modes, this kind of coupling will still occur because the actual springs stiffnesses are not ideal in coupling. The assumed drive displacement can easily reach above 10 μm, which magnitude is several orders larger than other sense modes. Under such circumstances, the cross coupling will be embodied from drive to sense modes.

**Figure 7:**
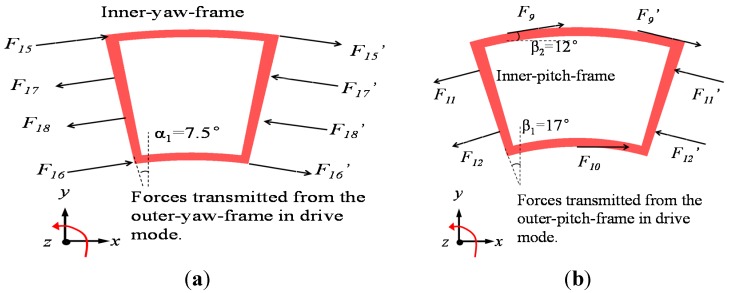
Mathematic models from drive to sense coupling effect: (**a**) Drive to yaw. (**b**) Drive to pitch/roll.

The coupling effects from drive-to-yaw and drive-to-pitch/roll are drawn in Figure 7a,b respectively. In pure mathematic modeling, both the inside yaw frame and pitch/roll frame have merely 1-DOF in their sense direction. Nevertheless, the stiffnesses of spring Nos. 9, 10, 15 and 16 in the drive direction are not infinite, which means that these decoupling springs are unavoidably subject to distortion in the drive axis via the force delivery from spring Nos. 11, 12, 17 and 18. Therefore, the sense frames will reveal undesirable rotational motion around *z*-axis, which will bring the coupling from drive mode to the sense modes. Supposing that the inside yaw frame and inside pitch/roll frame have an angular amplitude of *θ_1_* and *θ_2_* around the *z*-axis respectively, the mathematical model describing drive to sense coupling can be established by the force balance equations as:

(32) $$\left\{ \begin{array}{l} F_{x\_yaw}=2[\sum_{i=15,16}k_{i\_d}R_{i}{\text{θ}}_{1}\cos{\text{α}}_{1}-\sum_{j=17,18}k_{j}R_{j}(\text{θ}-{\text{θ}}_{1})\cos{\text{α}}_{1}]=0\\ F_{x\_pitch}=2k_{9\_d}R_{9}{\text{θ}}_{2}\cos{\text{β}}_{2}+k_{10\_d}R_{10}{\text{θ}}_{2}-2\sum_{i=11,12}k_{i}R_{i}(\text{θ}-{\text{θ}}_{2})\cos{\text{β}}_{1}=0 \end{array}\right.$$

where *F_x_yaw_ F_x_pitch_* are the forces in the *x*-axis of the inner-yaw-frame and inner-pitch-frame, respectively; *k_i_d_* (*I* = 9, 10, 15, 16) are the stiffnesses of spring Nos. 9, 10, 15 and 16 in the drive direction, respectively; *α_1_*, *β_1_*, *β_2_* are the included angles between the boundary of the frames/elastic forces and the *x/y*-axes, respectively.

Thus the drive-to-sense coupling ratios can be solved as:

(33) $$\left\{ \begin{array}{l} Couple_{d\_yaw}=\frac{{\text{θ}}_{1}}{\text{θ}}=\frac{\sum_{j=17,18}k_{j}R_{j}}{\sum_{i=15,16}k_{i\_d}R_{i}+\sum_{j=17,18}k_{j}R_{j}}=0.14\%\\ Couple_{d\_pitch}=\frac{{\text{θ}}_{2}}{\text{θ}}=\frac{2\sum_{i=7,8}k_{i}R_{i}\cos{\text{β}}_{1}}{2k_{9\_d}R_{9}{\text{θ}}_{2}\cos{\text{β}}_{2}+k_{10\_d}R_{10}+2\sum_{i=11,12}k_{i}R_{i}\cos{\text{β}}_{1}}=0.43\% \end{array}\right.$$

To verify the accuracy of the established drive-to-sense coupling model, the coupling effects are simulated by ANSYS static structural analysis. By applying a moment load on the drive frames around the *z*-axis, the coupling angular amplitude of the sense frames can be found by simulation. The simulation results of the drive-to-sense coupling effect are listed in Table 9. Obviously, the simulation results are in accordance with the theoretical analysis.

**Table 9:** Simulation results of drive-to-sense coupling.

Different Sense Modes	Drive Angular Amplitude (°)	Coupling Angular Amplitude (°)	Drive-to-Sense Coupling Ratio
Yaw	0.15	2.4 × 10^−4^	0.16%
Pitch/roll	0.15	7.1 × 10^−4^	0.47%

Similar to the drive-to-sense coupling simulation process, the sense mode coupling is easily obtained by ANSYS analysis. The simulated results tell us that the coupling effect between the sense modes are several orders of magnitude smaller, so they can be ignored in the analysis without any impact on the performance of the sense precision. Besides, the overlap areas for both yaw sense electrodes and pitch/roll electrode plates are bilateral structures, so they cannot be influenced by the drive mode motion. Thus, the changed capacitances are immune to drive to other modes coupling. In actual operation, a kind of dual channel closed-loop sense circuit will be adopted to suppress quadrature signals well [17]. For two second-order oscillators, if we adopt the open-loop sense circuit, indeed the frequencies cannot match ideally due to the cross-coupling term. However, if the cross-coupling term is suppressed successfully by a closed-loop strategy, the frequency can be matched well by further electrical trimming.

### 4.3. Sensitivity Analysis

**Figure 8:**
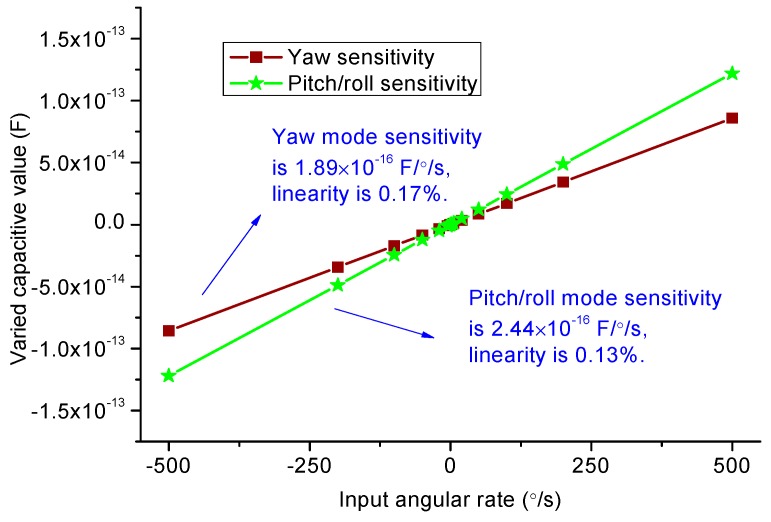
Sensitivity simulation results.

The gyroscope scale factor is calculated via harmonic response analysis of ANSYS when a range of input angular rates from 500 to 500°/s is exerted, the responsive motion in the sense frames is recorded subsequently. Through the transformation coefficients from the displacements to changed capacitance, scale factors for different sense modes can be fitted in Figure 8. The linearity indexes such as yaw mode and pitch/roll mode are about 0.17% and 0.13%, respectively. This scale factor nonlinearity mainly comes from the nonlinear expression of the spring stiffness. Thus the curve of Coriolis force (input angular rate) *versus* the displacement of the sensing element is nonlinear. A list of parameters such as Q factors and applied drive voltage values in simulation are given in Table 10. Note that the yaw mode Q factor is smaller than pitch or roll modes, the reason being that yaw mode detection has bigger electrode plate squeeze film damping. The sense modes mechanical Brownian noise is expressed at room temperature as [19]:

(34) $$Brown\_Noise=\frac{1}{A_{d}f_{d}}\sqrt{\frac{k_{B}Tf_{s}}{2\pi m_{s}Q_{s}}}$$

where *A_d_* is the average vibration amplitude of the sense mode frames around the *z*-axis; *k*_B_ = 1.38 × 10^−23^ J/K is the Boltzmann constant, *T* = 293 K is the absolute temperature; f_s_ is the sense mode frequency; *Q_s_* is the quality factor in sense mode; *m_s_* is the effective mass in sense mode. The simulation comparison with our precious triaxial linear type is summarized in Table 11. It is found that they have the same level of performance in sensitivity, linearity, and noise characteristics. Once we continue to further update its design, the corresponding performance will be improved. Considering that we need to find the trade-off between overall size and sensitivity for triaxial, we choose the working frequencies of 8 kHz here. Besides, to overcome the acoustic noise, we will adopt the encapsulation insulation technology in future.

**Table 10:** Some assumed parameters for the simulation.

Assumed Q-Factors in Different Modes			Assumed Drive Voltages (V)	
Drive (*Q_d_*)	Yaw (*Q_y_*)	Pitch/roll (*Q_p_*)	DC voltage	AC voltage
2000	500	1000	5	5

**Table 11:** Simulation result summary and comparison.

Simulation Results		Drive Mode	Yaw Mode	Pitch/Roll Mode
Natural frequency	Tri wheel	8001.1 Hz	8002.6 Hz	8002.8/8003.3 Hz
	Tri linear	14,017 Hz	14,018 Hz	14,020 Hz
Rotation angle/displacement sensitivity	Tri wheel	0.15° (around *z*-axis)	5.74 × 10^−10^ m/°/s	1.82 × 10^−5^ deg/°/s (around *x*/*y*-axes)
	Tri linear	6.44 × 10^−6^ m	4.07 × 10^−10^ m/°/s	7.62×10^−10^ m/°/s
Capacitance sensitivity	Tri wheel	3.76 × 10^−11^ F/°	1.89 × 10^−16^ F/°/s	2.44×10^−16^ F/°/s
	Tri linear	3.40 × 10^−8^ F/m	2.69 × 10^−16^ F/°/s	1.84×10^−16^ F/°/s
Sense linearity	Tri wheel	/	0.17%	0.13%
	Tri linear	/	0.12%	0.07%
Brownian noise floor	Tri wheel	/	0.32 °/h/√Hz	0.23 °/h/√Hz
	Tri linear	/	0.18 °/h/√Hz	0.17 °/h/√Hz

### 4.4. Fabrication Process

**Figure 9:**
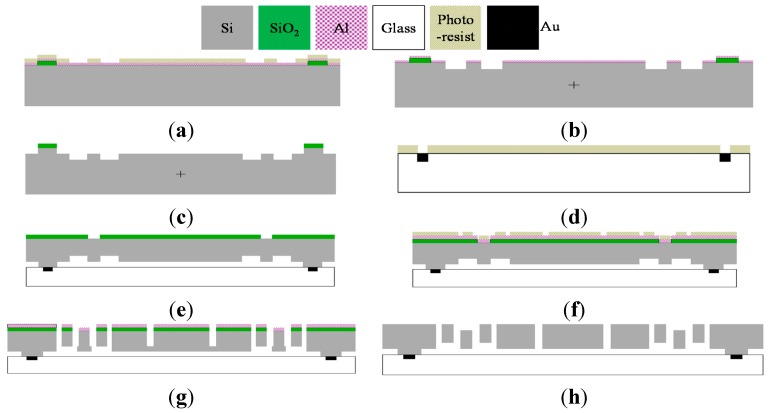
Fabrication process of the gyroscope. (**a**) Grow SiO_2_ by PECVD and Pattern Al lift-off; (**b**) Etch bottom trench by ICP; (**c**) Form anchor by ICP; (**d**) Etch Ti/Au by BOE and lift-off; (**e**) Define Upper trench by PECVD of SiO_2_; (**f**) Sputter Al by lift-off; (**g**) Release structure by ICP; (**h**) Etch upper trench by ICP.5.

The proposed triaxial gyroscope can be fabricated by the silicon-on-glass (SOG) process. The process can be divided into the eight steps as shown in Figure 9a–h.

The fabrication in Figure 9 begins with a (111) single crystal silicon wafer with low resistivity. Two layers of masks of SiO_2_ and Al should be patterned by PECVD and lift-off processes, respectively, which are responsible for defining the anchor mask and bottom trench mask (Figure 9a). Then the trenches are first etched 15 μm depth by ICP etching (Figure 9b) and next an anchor of 40 μm height is formed by the second ICP etching (Figure 9c). Besides, a Pyrex 7740 glass layer is prepared to define the Ti/Au composite electrodes by BOE etching and lift off process (Figure 9d). The above two wafer are Si glass anodic bonded together. The silicon wafer will be thinned to 100μm by CMP and similarly coated by PECVD etched SiO_2_ to define the upper trench of 15 μm (Figure 9e). Next, Al is sputtered on the SiO_2_ to define the structure pattern by lift off again (Figure 9f). Finally, the upper trenches are first ICP etched 45 μm depth to almost release the structure (Figure 9g) and then ICP etched 15 μm to generate the precise trench depth and etch through the device fully (Figure 9h). The vacuum encapsulation will be finished via a metallic package with special getter materials. At the same time all the electrodes will be wire bonded out to realize the electrical functions for testing.

## 5. Conclusions and Future Work

In this work, we present a new triaxial vibratory wheel gyroscope with fully decouped functions. The cross-decoupling effects between drive and other sense modes have been eliminated by elaborately arranged springs. The arrangements and dimensions of all the rings and frames are determined by carefully calculating the drive force, sensitivities and masses in different modes. Then the AFSA is adopted to determine the springs’ parameters to quickly process the mode match. After this optimization operation, the frequency splits between the drive and sense modes can reach a proper gap of 108 Hz. Afterwards, this frequency gap is reduced to below 3 Hz by repeatedly trimming a number of key springs sizes. Moreover, the stiffness tuning electrodes are designed in all the sense modes, so that the frequency split can be minimized by applying a proper DC voltage to the tuning electrodes after fabrication. The simulation results in ANSYS show that the coupling magnitudes among drive and other sense modes are below 0.2%. The yaw and pitch/roll modes scale factors are 0.189 fF/°/s and 0.244 fF/°/s under the *Q* values of 500 and 1000 respectively.

## References

[1] Liu K., Zhang W., Chen W., Li K., Dai F., Cui F., Wu X., Ma G., Xiao Q. (2009). The development of micro-gyroscope technology. J. Micromech. Microeng..

[2] Xia D.Z., Yu C., Kong L. (2013). A micro dynamically tuned gyroscope with adjustable static capacitance. Sensors.

[3] Xia D., Kong L., Hu Y., Ni P. (2015). Silicon microgyroscope temperature prediction and control system based on BP neural network and Fuzzy-PID control method. Meas. Sci. Technol..

[4] Xia D., Yu C., Kong L. (2014). The Development of Micromachined Gyroscope Structure and Circuitry Technology. Sensors.

[5] Kim J., Park S., Kwak D., Ko H., Cho D. (2005). An *x*-axis single-crystalline silicon microgyroscope fabricated by the extended SBM process. J. Microelectromech. Syst..

[6] Guo Z., Yang Z., Zhao Q., Lin L.T., Ding H.T., Liu X.S., Cui J., Xie H., Yan G.Z. (2010). A lateral-axis micromachined tuning fork gyroscope with torsional Z-sensing and electrostatic force-balanced driving. J. Micromech. Microeng..

[7] Zhao Q., Lin L., Yang Z., Dong L., Yan G. A micromachined vibrating wheel gyroscope with folded beams. Proceedings of the 2013 IEEE Sensors.

[8] ST Microelectronics L3G4200D-3-Axis Digital Output Gyroscope. http://www.st.com.

[9] Li J., Broas M., Makkonen J., Mattila T.T., Hokka J., Paulasto-Krockel M. (2014). Shock impact reliability and failure analysis of a three-axis MEMS gyroscope. J. Microelectromec. Syst..

[10] Walther A., Desloge B., Lejuste C., Coster B., Audebert P., Willienmin J. (2013). Development of a 3D capacitive gyroscope with reduced parasitic capacitance. J. Micromech. Microeng..

[11] Vigna B. Tri-axial MEMS gyroscopes and Six Degree-Of-Freedom Motion Sensors. Proceedings of the 2011 IEEE Electron Devices Meeting (IEDM).

[12] Tsai N., Sue C. (2010). Experimental analysis and characterization of electrostatic-drive tri-axis micro-gyroscope. Sens. Actuators A.

[13] Wang M., Jiao J., Yan P., Mi B.W., Qi S. (2014). A novel tri-axis MEMS gyroscope with in-plane tetra-pendulum proof masses and enhanced sensitive springs. J. Micromech. Microeng..

[14] Geiger W., Butt W., Gainer A., Frech J., Braxmaier M., Link T., Kohne A., Nommensen P., Sandmaier H., Lang W. (2002). Decoupled microgyros and the design principle DAVED. Sens. Actuators A.

[15] Alper S., Akin T. (2005). A single-crystal silicon symmetrical and decoupled MEMS gyroscope on an insulating substrate. J. Microelectromec. Syst..

[16] Zotov S., Trusov A., Shkel A. (2012). High-range angular rate sensor based on mechanical frequency modulation. J. Microelectromec. Syst..

[17] Xia D., Kong L., Gao H. (2015). Design and Analysis of a Novel Fully Decoupled Tri-axis Linear Vibratory Gyroscope with Matched Modes. Sensors.

[18] Fowles G., Cassiday G. (1999). Analytical Mechanics.

[19] Sharma A., Zaman M., Ayazi F. (2009). A sub-0.2°/h bias drift micromechanical silicon gyroscope with automatic CMOS mode-matching. IEEE J. Solid-State Circuits.

[20] Fedder G.K. (1994). Simulation of Microelectromechanical Systems. Ph.D. Thesis.

[21] Zhang Z., Wang G., Zou K., Zhang J. (2014). A Solution Quality Assessment Method for Swarm Intelligence Optimization Algorithms. Sci. World J..

[22] Shen M., Li L., Liu D. Research and Application of Function Optimization Based on Artificial Fish Swarm Algorithm. Proceedings of the 4th International Conference on Computer Engineering and Networks (CENet2014).

[23] Neshat M., Sepidnam G., Sargolzaei M., Toosi A.N. (2014). Artificial fish swarm algorithm: A survey of the state-of-the-art, hybridization, combinatorial and indicative applications. Artif. Intell. Rev..

